# A small molecule, (E)-2-methoxy-4-(3-(4-methoxyphenyl) prop-1-en-1-yl) phenol suppresses tumor growth via inhibition of IkappaB kinase β in colorectal cancer *in vivo* and *in vitro*

**DOI:** 10.18632/oncotarget.20440

**Published:** 2017-08-24

**Authors:** Jie Zheng, Mi Hee Park, Hee Pom Lee, Byung Kook Hyun, Hyung Ok Chun, Sung Hee Jung, Hyun Ok Seo, Young Wan Ham, Sang-Bae Han, Jin Tae Hong

**Affiliations:** ^1^ College of Pharmacy & Medical Research Center, Chungbuk National University, Cheongju, Chungbuk 28160, Republic of Korea; ^2^ Department of Chemistry, Utah Valley University 800 W, University Pkwy, Orem, UT 84058, USA; ^3^Current address: Tumor Microenvironment Global Core Research Center, College of Pharmacy, Seoul National University, Seoul, 08826, Republic of Korea

**Keywords:** MMPP, colon cancer, IKKβ, death receptor, tumor growth inhibition

## Abstract

Here we report that a novel synthesized compound (E)-2-methoxy-4-(3-(4-methoxyphenyl)prop-1-en-1-yl)phenol (MMPP) which exhibits better stability, drug-likeness and anti-cancer effect than (E)-2,4-bis(p-hydroxyphenyl)-2-butenal (BHPB) that we previously reported. Of all newly synthesized BHPB analogues, MMPP showed the most significant inhibitory effect on colon cancer cell growth. Thus, we evaluated the anti-cancer effects and possible mechanisms of MMPP *in vitro* and *in vivo*. MMPP treatment (0-15 μg/mL) induced apoptotic cell death and enhanced the expression of cleaved caspase-3 and cleaved caspase-8 in a concentration dependent manner. Notably, the expression of death receptor (DR)5 and DR6 was significantly increased by MMPP treatment. Moreover, DR5 siRNA or DR6 siRNA transfection partially abolished MMPP-induced cell growth inhibition. Pull down assay and docking experiment showed that MMPP bound directly to IkappaB kinase β (IKKβ). It was noteworthy that IKKβ mutant (C99S) partially abolished MMPP-induced cell growth inhibition and enhanced expression of DR5 and DR6. In addition, MMPP enhanced TRAIL-induced apoptosis, cell growth inhibition and expression of DRs. In xenograft mice model, MMPP (2.5-5 mg/kg) suppressed tumor growth in a dose dependent manner. Immunohistochemistry analysis showed that the expression levels of DR5 and DR6 and active caspase-3 were increased while the expression levels of PCNA and p-IKKβ were decreased in a dose dependent manner. Thus, MMPP may be a promising anti-cancer agent in colon cancer treatment.

## INTRODUCTION

Colorectal cancer (CRC, also known as colon cancer) is the third common cancer and the third leading cause of cancer death in both men and women [1]. About 54 percent of colorectal cancer cases occurred in more developed countries. The highest incidence of colorectal cancer was in Oceania and Europe, and the lowest incidence in Africa and Asia. But Republic of Korea had the highest rate of colorectal cancer in recent years [2]. As present treatments for colon cancer patients are not so sufficient, it is urgent to develop appropriate novel chemo-preventive compounds with less toxicity.

Nuclear factor kappa-light-chain-enhancer of activated B cells (NF-κB) plays a vital role on cell survival via inhibition of apoptosis and contribution of cancer development [3]. NF-κB is constitutively activated in various human cancer tissues and cell lines including colorectal cancer [4, 5]. In general, NF-κB signaling pathway is triggered by activation of IKK complex [6]. Phosphorylation of IκBα mainly depends on the IκB kinase β (IKKβ) catalytic subunit of the IKK complex [6]. Greten *et al.* found that deletion of IKKβ in intestinal epithelial cells in a mouse model of colitis-associated model for cancer (CAC) dramatically reduced tumor number [7]. Moreover, impaired NF-κB activation was observed in IKKβ-deficient mice [8]. Thus, most chemopreventive agents suppressing NF-κB by targeting IKKβ may show significant anti-cancer effect in colorectal cancer [9]. In our previous study, (E)-2,4-bis(p-hydroxyphenyl)-2-butenal (BHPB) suppressed colon tumor growth via inhibition of NF-κB signaling pathway by targeting IKKβ [10].

Apoptosis can be induced through the activation of death receptors (DRs) such as Fas, DR3, DR4, DR5 and DR6 binding with each ligand [11-13]. These interactions induce apoptosis through activation of caspase families [12]. Recruitment of caspase-8 through Fas associated death domain (FADD) leads to its acute-cleavage and activation [14, 15], and active caspase-8 in turn activates effector caspases such as caspase-3 causing the cell to undergo apoptosis [14, 16]. DR5 knockout mice are compromised in radiation induced apoptosis [17]. Activation of DRs could enhance susceptibility of cancer cells toward chemotherapeutics [18]. Numerous studies have demonstrated that DR signaling was correlated with cell death in colorectal cancer. It was previously found that bee venom, snake venom toxin, tectochrysin induced apoptosis via up-regulation of DRs in colon cancer cells [19-21]. These findings indicate that diverse activation of DRs depends on chemical properties.

Recently, we have found that BHPB showed anti-cancer effect [10, 22]. However, the existence of aldehyde functionality made the compound lack stability and drug-likeness. To overcome the roadblock, we synthesized several BHPB analogues by Heck reaction which have more stable and proper drug-likeness properties than BHPB. We selected one analogue (E)-2-methoxy-4-(3-(4-methoxyphenyl) prop-1-en-1-yl) phenol (MMPP), and evaluated the anti-cancer mechanisms and effects of MMPP on colon cancer cell growth *in vivo* and *in vitro*.

## RESULTS

### MMPP inhibits colon cancer cell growth

**Figure 1:**
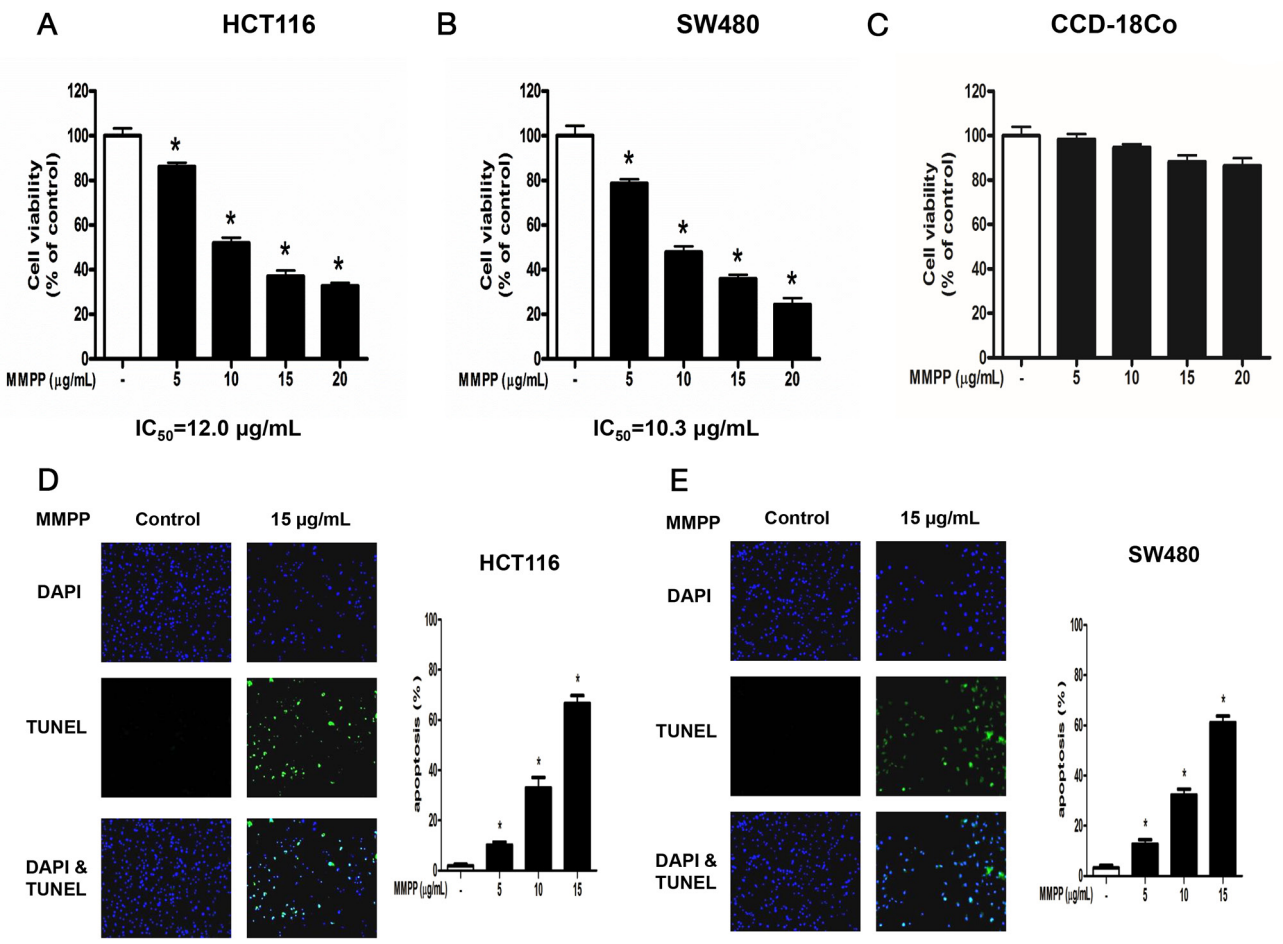
Effect of MMPP on the growth colon cancer cells and colon epithelial normal cells Concentration-dependent inhibitory effect of MMPP on cancer cell growth was found in HCT116 and SW480 colon cancer cells but not in CCD-18Co colon epithelial normal cells. **(A)**, **(B)** & **(C)** HCT116, SW480 and CCD-18Co cells were treated with MMPP (0-20 μg/mL) for 24 h, and then relative cell survival rate was determined by MTT assay. Data was expressed as the mean ± S.D. of three experiments. *p<0.05 indicates significant difference from control group. **(D)** & **(E)** Apoptotic cell death of HCT116 and SW480. Colon cancer cells were treated with MMPP (0-15 μg/mL) for 24 h, and then labeled with DAPI and TUNEL solution. Total number of cells in a given area was determined by using DAPI nuclear staining (fluorescent microscope). A green color in the fixed cells marks TUNEL-labeled cells. Apoptotic index was determined as the DAPI-stained TUNEL-positive cell number /total DAPI-stained cell number x 100%. Data was expressed as the mean ± S.D. of three experiments. *p<0.05 indicates significant difference from control group.

Cell viability was assessed by using MTT assay to evaluate the effect of MMPP on colon cancer cells. MMPP (0-20 μg/mL or 0-74 μM) inhibited colon cancer cell growth and IC_50_ values on HCT116 (Figure 1A) and SW480 (Figure 1B) were 12.00 μg/mL (44.39 μM) and 10.30 μg/mL (38.10 μM), respectively. However, in our previous study, IC_50_ of BHPB on HCT116 was 25 μg/mL (98.31 μM). Notably, MMPP exhibited little toxicity on CCD-18Co colon epithelial normal cells (Figure 1C).

### MMPP induces apoptosis and regulates apoptosis-associated protein expression

TUNEL assay was performed to determine whether the cell growth inhibition by MMPP was due to induction of apoptosis. The cells were treated with MMPP (0-15 μg/mL) for 24 h, DAPI-stained TUNEL-positive cells were concentration-dependently increased and the highest concentration of MMPP (15 μg/mL) caused most of cells TUNEL-positive, and apoptosis rates were 67.95 % in HCT116 cells (Figure 1D) and 61.26 % in SW480 cells (Figure 1E).

**Figure 2:**
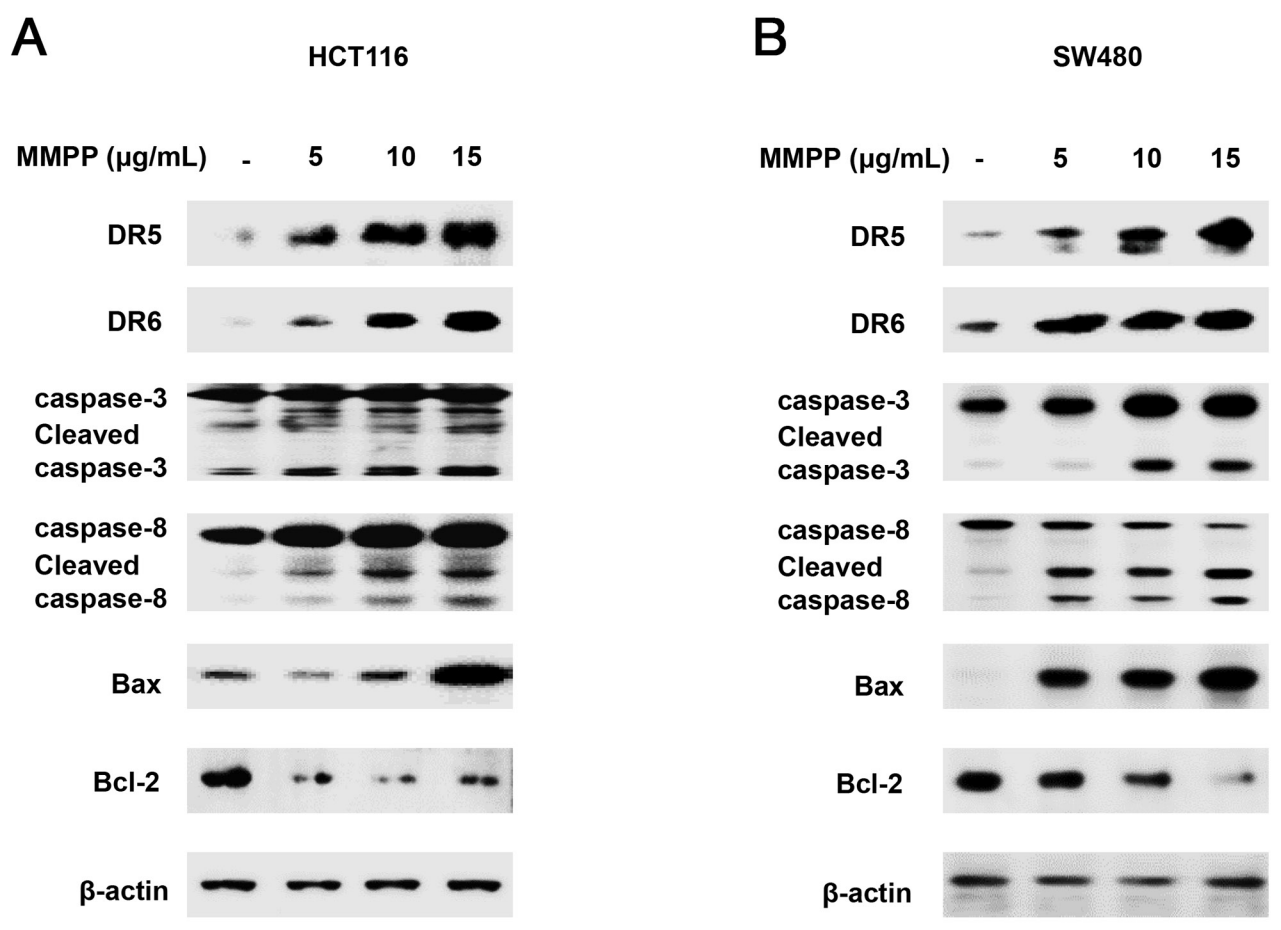
Effect of MMPP on apoptosis regulatory proteins in colon cancer cells **(A)** & **(B)** Expression of apoptosis regulatory proteins was determined by Western blot analysis with antibodies against DR5, DR6, cleaved capase-3, cleaved caspase-8, Bax, Bcl-2 and β-actin (internal control). Each band is representative for three experiments.

Next, the expression of apoptotic proteins was investigated. MMPP (0-15 μg/mL) enhanced the expression of apoptotic proteins such as Bax, cleaved caspase-3 and cleaved caspase-8 while decreased the expression of anti-apoptotic protein Bcl-2 in HCT116 (Figure 2A) and SW480 (Figure 2B) colon cancer cells. These findings suggested that MMPP strongly induced apoptosis in HCT116 and SW480 colon cancer cells.

### MMPP directly binds to IKKβ

**Figure 3:**
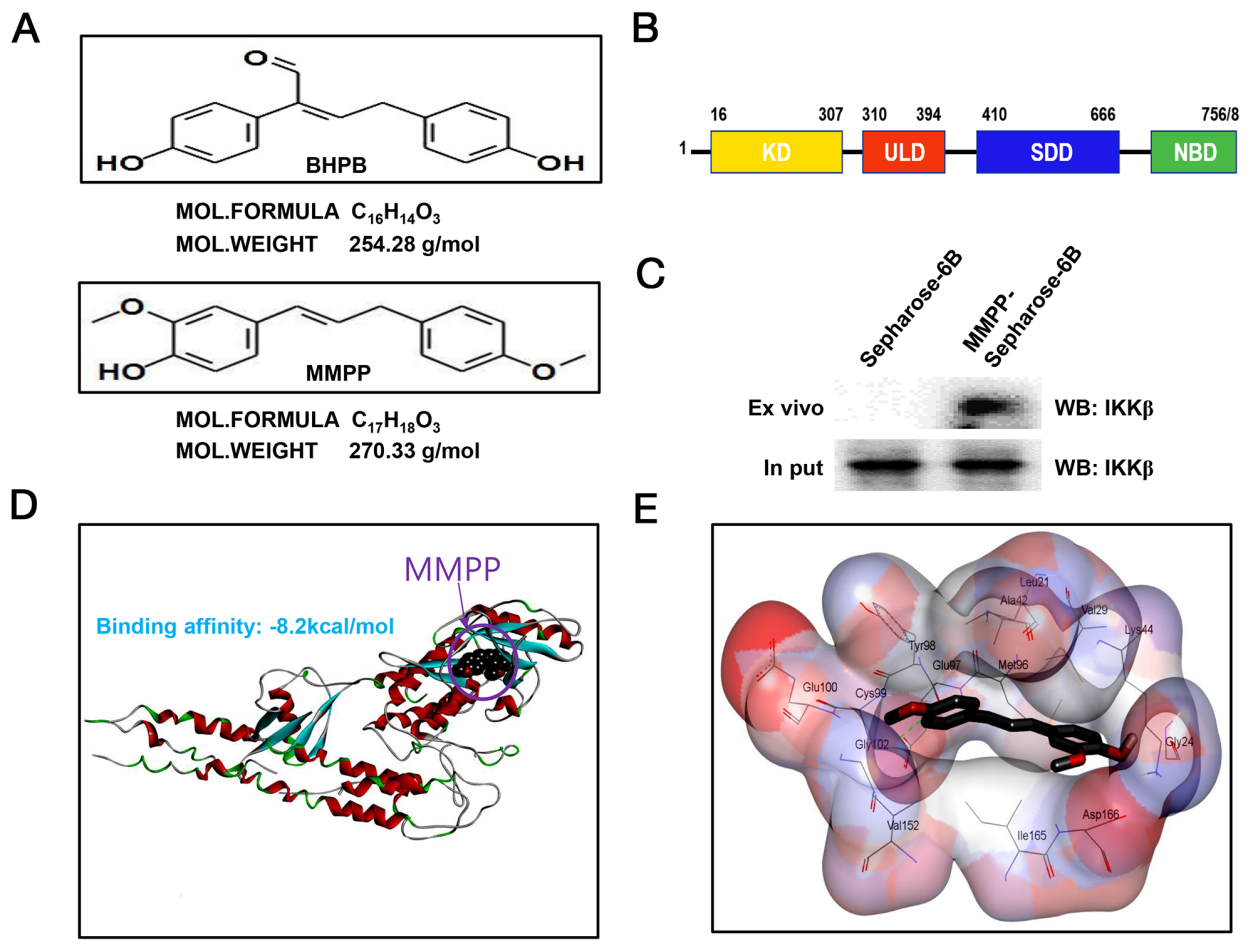
Structure of MMPP, and molecular binding between MMPP and IKKβ **(A)** (up) Structure of BHPB. (bottom) Structure of MMPP. **(B)** Structure of IKKβ. **(C)** Pull-down assay identifies molecular binding between MMPP and IKKβ. MMPP was conjugated with epoxy-activated Sepharose 6B. **(D)** & **(E)** Docking model of MMPP with IKKβ was performed as described in the materials and methods.

Structures of BHPB and MMPP were showed with Medchem designer 3.0 (Figure 3A). Structure of IKKβ was presented (Figure 3B). The molecular binding between MMPP and IKKβ was assessed by pull-down assay. The binding of MMPP-Sepharose 6B beads with IKKβ was then detected by immunoblotting with IKKβ antibody. The results indicated that MMPP interacted with cell lysates containing IKKβ (Figure 3C). Molecular docking experiment revealed that MMPP directly bound to the ATP binding site of IKKβ with a strong binding affinity (-8.2 kcal/mol) (Figure 3D). Concretely, MMPP bound to hydrophobic binding pocket composed of Leu21, Val29, Tyr98, Cys99, Val152 and lleq65 (Figure 3E).

### MMPP suppresses phosphorylation of IKKβ

**Figure 4:**
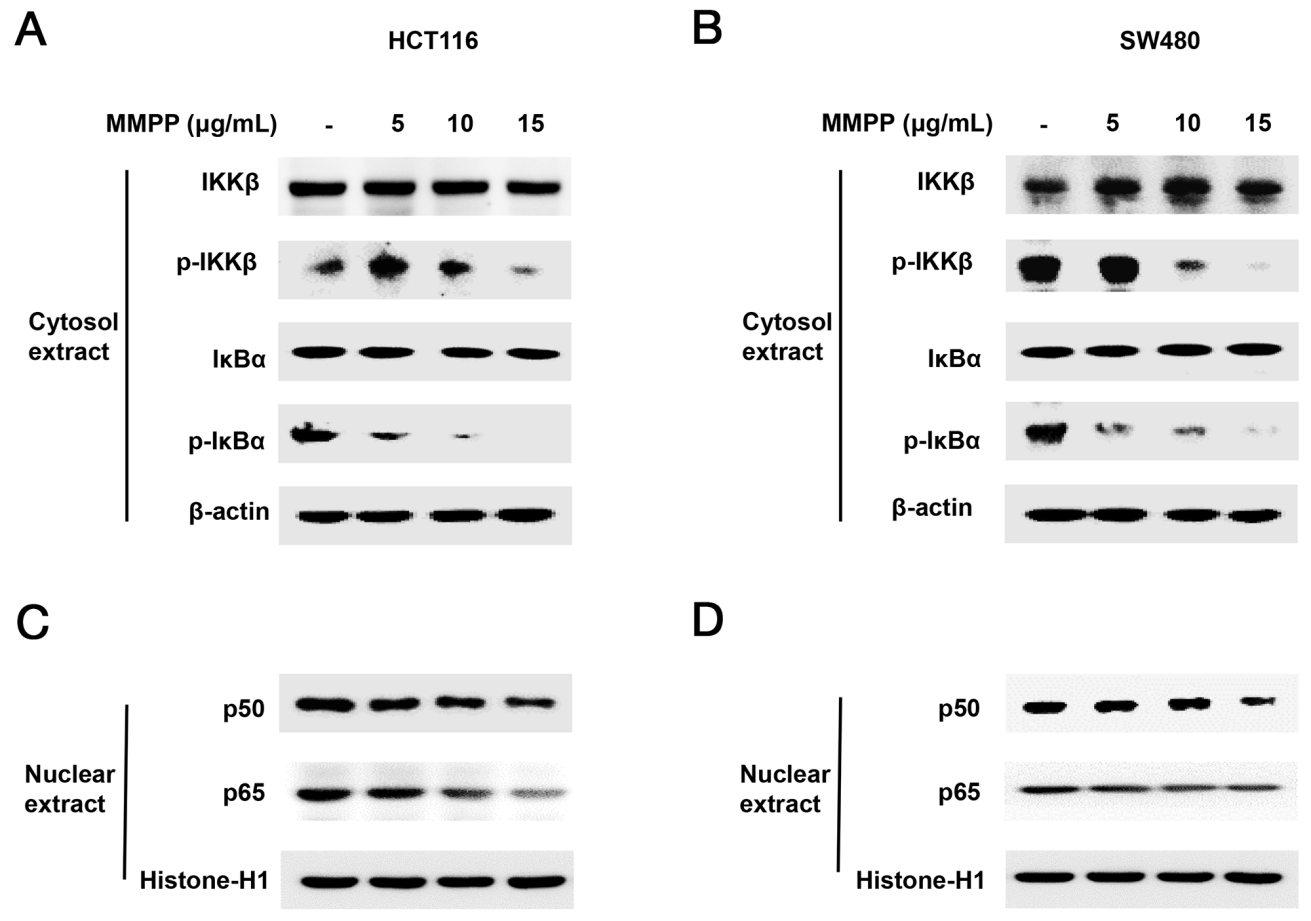
Effect of MMPP on IKKβ-dependent NF-κB activation Colon cancer cells were treated with MMPP (0-15 μg/mL) for 2 h, and then were lysed with A buffer and C buffer. **(A)** & **(B)** Cytosol proteins were used to determine the expression of IKKβ, p-IKKβ, IκBα, p-IκBα and β-actin (internal control). **(C)** & **(D)** Nuclear proteins were used to determine the expression of, p50, p65 and Histone H1 (internal control) in colon cancer cells. Each band is representative for three experiments.

We performed Western blotting to investigate whether MMPP suppresses phosphorylation of IKKβ which is one of the main NF-κB upstream signals. The results showed that phosphorylation of IKKβ and IκBα were decreased (Figure 4A & 4B), and nuclear translocation of p50 and p65 were decreased in a concentration dependent manner in HCT116 (Figure 4C) and SW480 colon cancer cells (Figure 4D).

### Abrogated effect of IKKβ mutant C99S with MMPP on cell growth and DR expression

**Figure 5:**
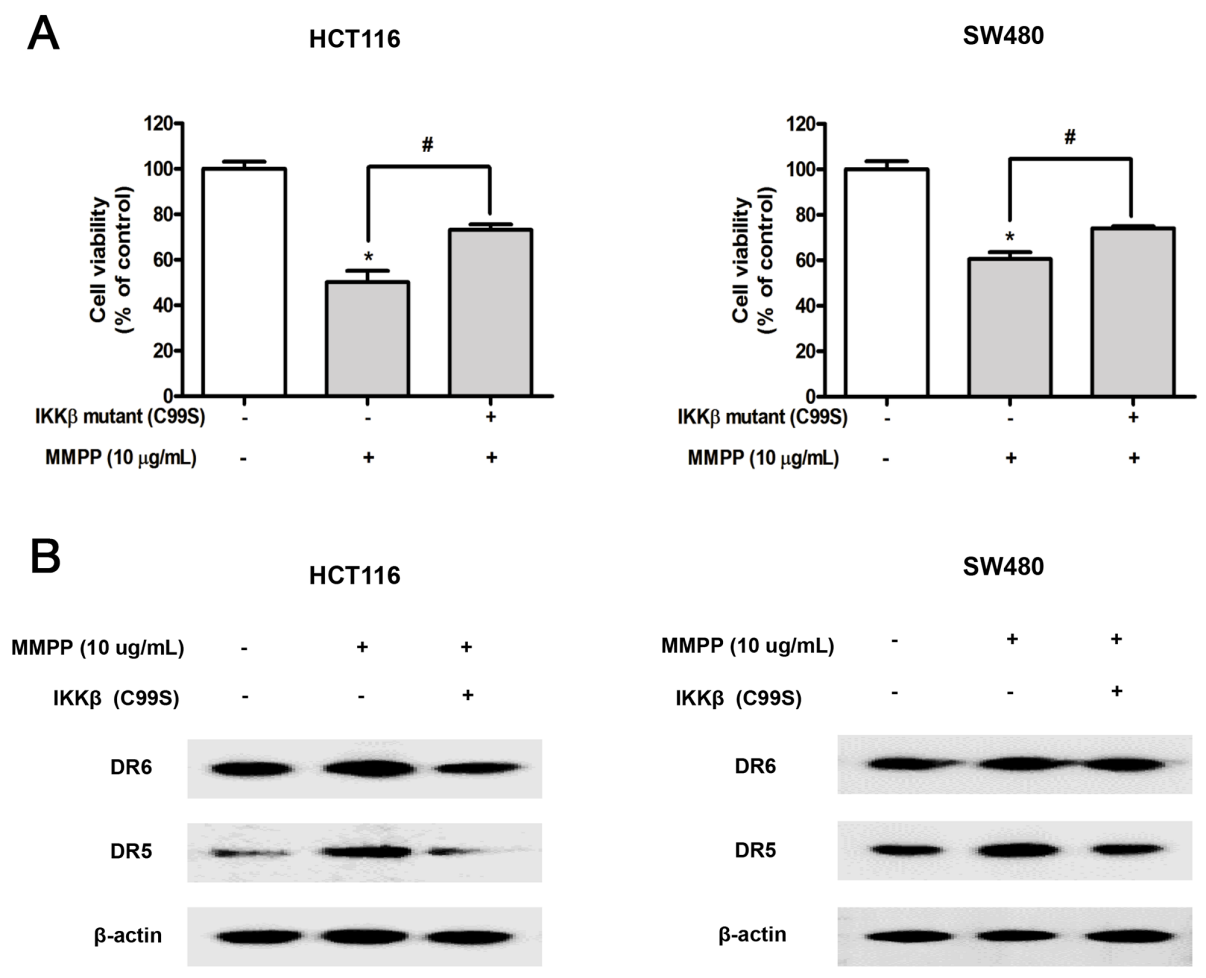
Effect of IKKβ mutant (C99S) on MMPP-induced inhibitory effect of cell growth, enhanced expression of DRs **(A)** Cells were transfected with IKKβ mutant (C99S) for 48 h, and then treated with MMPP (10 μg/mL) for 24 h. Relative cell survival rate was determined by MTT assay. Data was expressed as the mean ± S.D. of three experiments. *p<0.05 indicates significant difference from control group. **(B)** Protein expression was determined with Western blotting with antibodies against DR5, DR6 and β-actin (internal control).

To further investigate the role of IKKβ on the anti-cancer effect of MMPP, the effect of IKKβ mutant C99S was investigated. Both cells were transfected with IKKβ mutant C99S for 24 h, and then treated with MMPP for 24 h to assess cell viability and protein expression. MMPP-induced inhibitory effect on cell growth was partially abolished (Figure 5A) and MMPP-induced enhanced expression of DR5 and DR6 was partially abrogated (Figure 5B).

### Synergistic effect of TNF-related apoptosis-inducing ligand (TRAIL) with MMPP on cell growth and expression of DRs

**Figure 6:**
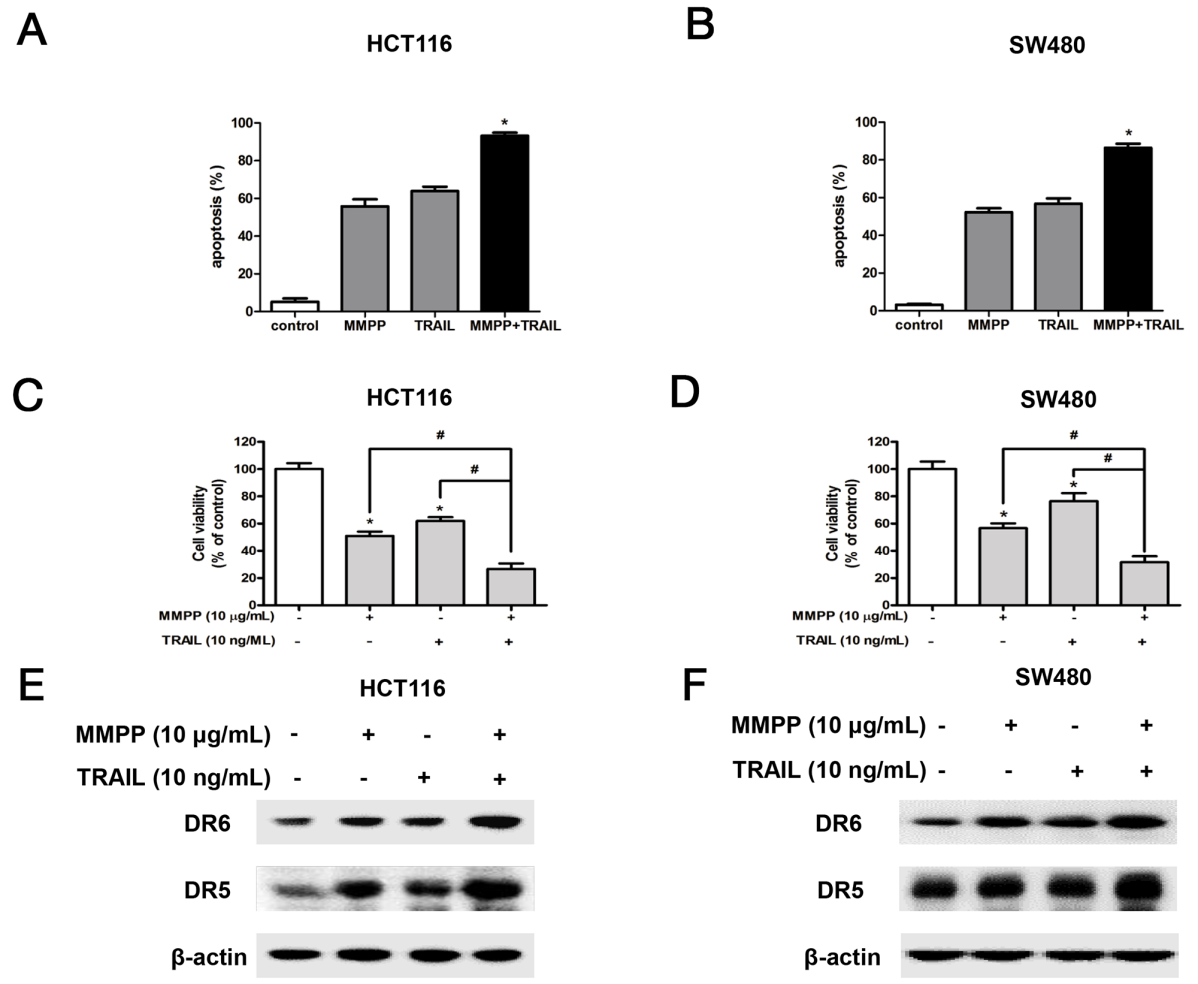
Effect of co-treatment with TRAIL and MMPP on apoptosis, cell growth and expression of DRs **(A)** & **(B)** Colon cancer cells were pre-treated with TRAIL recombinant protein (10 ng/mL) for 2 h, and then treated with MMPP (10 μg/mL) for 24 h. Apoptotic index was determined as the DAPI-stained TUNEL-positive cell number /total DAPI-stained cell number x 100%. Data was expressed as the mean ± S.D. of three experiments. *p<0.05 indicates significant difference from control group. **(C)** & **(D)** Relative cell survival rate was determined by MTT assay. Data was expressed as the mean ± S.D. of three experiments. *p<0.05 indicates significant difference from control group. **(E)** & **(F)** Protein expression was determined with Western blotting with antibodies against DR5, DR6 and β-actin (internal control).

To investigate whether MMPP could enhance TRAIL induced apoptosis and cell growth inhibition, both cells were pre-treated with TRAIL for 2 h, and treated with MMPP (10 μg/mL) for 24 h to assess apoptosis, cell viability and protein expression. As a result, MMPP augmented TRAIL-induced apoptosis (Figure 6A & 6B), cell growth inhibition (Figure 6C & 6D), enhanced the expression of DR5 and DR6 (Figure 6E & 6F) in HCT116 and SW480 colon cancer cells.

### MMPP suppresses tumor growth in colon cancer xenograft model

**Figure 7:**
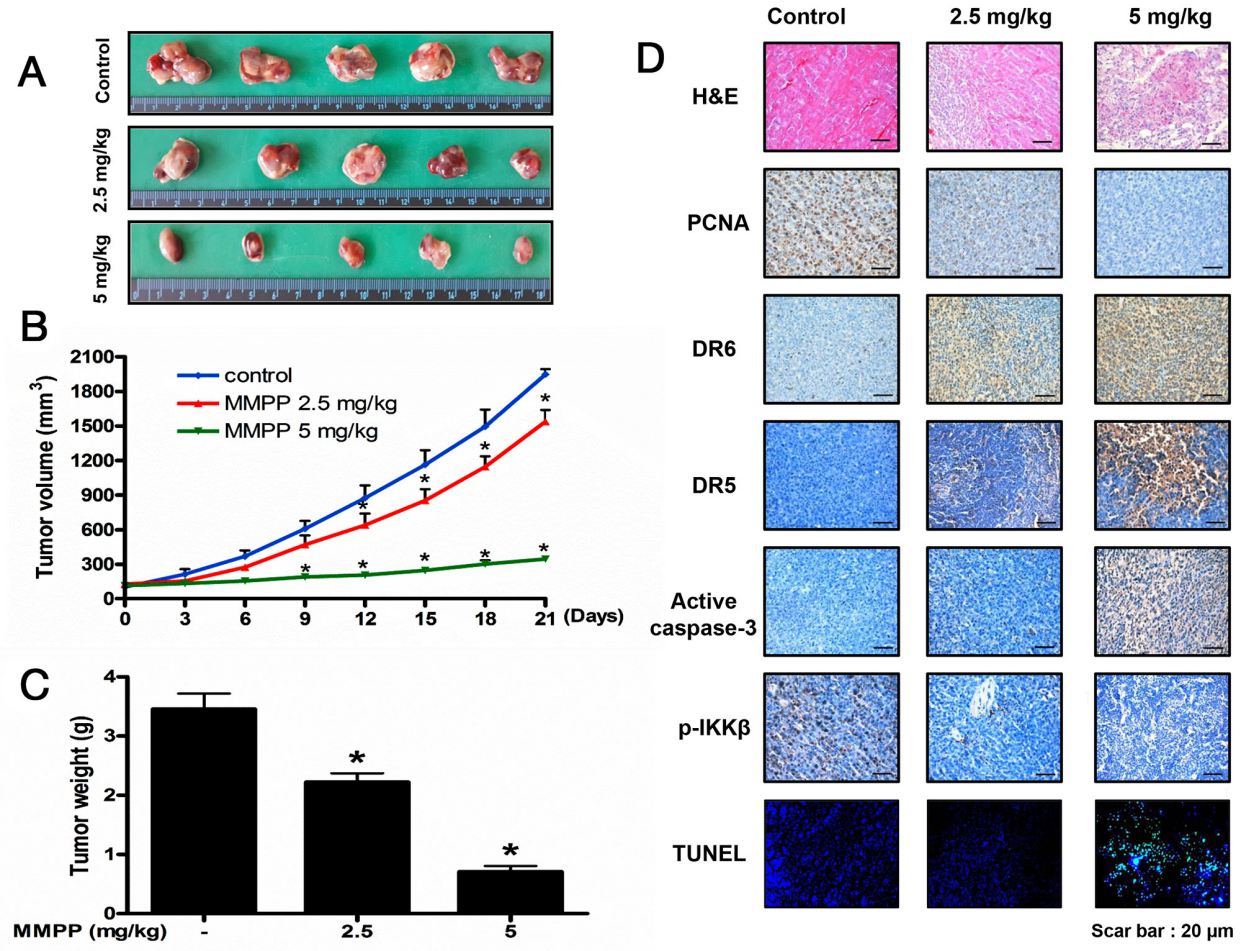
Anti-tumor activity of MMPP in colon cancer xenograft mice model **(A)**, **(B)** & **(C)** Growth inhibition of subcutaneously transplanted HCT116 xenograft mice treated with MMPP (2.5 mg/kg and 5 mg/kg twice a week) for 3 weeks. Xenograft mice (n=10) were administrated intraperitoneally with 0.01% DMSO or MMPP (2.5 mg/kg and 5 mg/kg). Tumor burden was measured once per week using a caliper, and calculated volume length (mm) × width (mm) × height (mm)/2. Tumor weight and volume are presented as means ± S.D. **(D)** Immunohistochemistry was used to determine expression levels of PCNA, DR6, DR5, active caspase-3, p-IKKβ in nude mice xenograft tissues by the different treatments as described in the materials and method. DAPI&TUNEL assay was carried out to assess the apoptosis rate in the nude mice xenograft tissue. Total number of cells in a given area was determined by using DAPI nuclear staining (fluorescent microscope). A green color in the fixed cells marks TUNEL-labeled cells.

To demonstrate the anti-cancer effect of MMPP *in vivo*, the tumor growth on HCT116 xenograft nude mice following MMPP treatment was investigated. In HCT116 xenograft studies, MMPP (2.5-5 mg/kg) was administrated intraperitoneally twice per week for 3 weeks to mice with tumors ranging from 100-150 mm^3^. All mice were sacrificed at the end of experiment when tumors were dissected and weighted. The inhibitory effect of MMPP on tumor growth was significant in xenograft mice model (Figure 7A). Tumor volume and tumor weight were dose-dependently decreased (Figure 7B & Figure 7C). The immunohistochemistry analysis revealed that MMPP dose-dependently suppressed tumor growth, and enhanced the expression levels of DR5, DR6 and active-caspase-3 while decreased the expression levels of p-IKKβ in nude mice xenograft tissues (Figure 7D).

### Effect of MMPP on the expression of apoptosis regulatory proteins in colon xenograft tumor tissues

**Figure 8:**
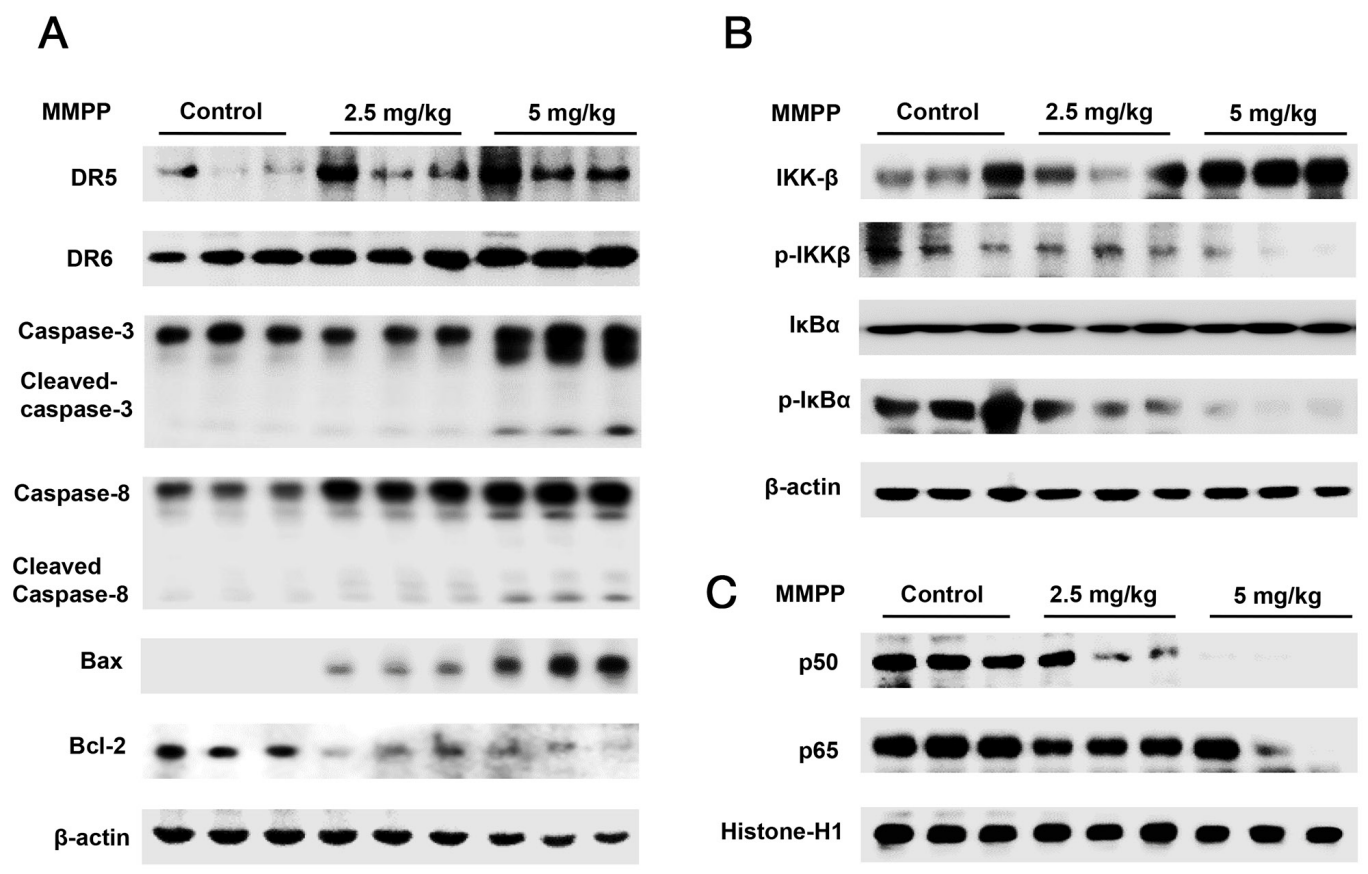
Effect of MMPP on the expression of apoptosis regulatory proteins, NF-κB associated and subunit proteins **(A)** Expression of apoptosis regulatory proteins was determined by Western blot analysis with antibodies against DR5, DR6, cleaved capase-3, cleaved caspase-8, Bax, Bcl-2 and β-actin (internal control). **(B)** Cytosol proteins were used to determine the expression of IKKβ, p-IKKβ, IκBα, p-IκBα and β-actin (internal control). **(C)** Nuclear proteins were used to determine the expression of, p50, p65 and Histone-H1 (internal control) in xenograft tumor tissues. Each band is representative for three experiments.

Western blotting was carried out to examine the relationship between tumor growth and apoptosis regulatory proteins, as well as NF-κB activity. We found that the expression of cleaved caspase-3, cleaved caspase-8 and Bax was increased in a dose dependent manner (2.5-5 mg/kg) while the expression of Bcl-2 was decreased (Figure 8A). We also found the phosphorylation of IKKβ, IκBα and the translocation of p50 and p65 were decreased in a dose dependent manner (2.5-5 mg/kg) (Figure 8B & 8C) in colon xenograft tumor tissues.

## DISCUSSION

In the present study, MMPP suppressed tumor growth of colon cancer in a dose dependent manner (2.5-5 mg/kg) via up-regulation of DR5, DR6 and inhibition of IKKβ/NF-κB activity in HCT116 xenograft nude mice model. In addition, MMPP inhibited colon cancer cell growth by apoptosis induction, and inhibition of IKKβ/NF-κB in HCT116 and SW480 colon cancer cells *in vitro*. These effects were correlated with the enhanced expression of various apoptotic proteins such as Bax, cleaved caspase-3 and cleaved caspase-8. However, the expression of anti-apoptotic proteins such as Bcl-2 was decreased. Notably, the expression of DRs such as DR5 and DR6 was dramatically increased by MMPP concentration-dependently.

Tumor tissues obtained from colon cancer patients showed extremely high levels of NF-κB activity [23]. Suppressing NF-κB with diverse approaches such as siRNA, IKK inhibitors and IκBα suppressor inhibited survival and proliferation of cancer cells [24]. Suppression of NF-κB involves targeting various components or steps in the NF-κB signaling pathway such as inactivation of IKK, degradation of IκBα, nuclear translocation and DNA binding activity of NF-κB [24-26]. Among these targets, IKK was thought to be the most effective and selective drug target [27]. Various studies have proposed that inhibition of NF-κB signaling pathway by targeting IKKβ may play a vital survival role in colon cancer. A limonoid triterpene, Nimbolide inhibits growth of human colon cancer xenografts by inhibition of IKK activation [28]. (E)-2,4-bis(p-hydroxyphenyl)-2-butenal suppressed colon tumor growth via inhibition of NF-κB by targeting IKKβ [10]. Similarly, the present data showed that MMPP suppressed colon tumor growth via targeting IKKβ. It was noteworthy that molecular docking experiment revealed that MMPP directly bound to the ATP binding site of IKKβ, hydrophobic binding pocket composed of Leu21, Val29, Tyr98, Cys99, Val152 and lleq65. Interestingly, the binding affinity of MMPP and IKKβ was -8.2 kcal/mol, and the binding domain was the same location as the known inhibitor [29]. However, in our previous study, the binding affinity of BHPB and IKKβ was -7.3 to -8.0 kcal/mol. This finding indicated that more stable structure contributed to the higher binding affinity with IKKβ. In addition, pull down assay also showed that MMPP directly bound to IKKβ recombinant protein. In addition, MMPP-induced inhibitory effect on cell growth was partially abolished in C99S mutated IKKβ. This may be because MMPP could not bind to C99S mutated IKKβ, thereby exhibiting no significant anti-cancer effect. Therefore, IKKβ/NF-κB signaling plays a critical role on MMPP-induced cell growth inhibition and apoptotic cell death.

Inhibition of NF-κB by blocking activation of IKK sensitizes tumor cells to TRAIL-induced apoptosis [30]. TRAIL selectively induces apoptosis of a variety of tumor cells and transformed cells, but not most normal cells [31]. Present findings showed that co-treatment with TRAIL and MMPP enhanced TRAIL-induced apoptosis and cell growth inhibition, and magnified the expression of DR5 and DR6. These effects were found to be synergistic because combination index (CI) value was evaluated and found smaller than 1. These data indicated that inhibitory effect on IKK/NF-κB signaling by MMPP could enhance TRAIL-induced apoptosis. Notably, inhibition of NF-κB by several chemotherapeutics exposures human cancer cells to induction of apoptosis through activation of DRs [32]. Recent studies on the signaling mechanisms of DR have revealed that members of the NF-κB are key regulators of apoptotic cell death [33]. Moreover, it was also well-documented that NF-κB directly up-regulates anti-apoptotic proteins such as C-FLIP which is a specific inhibitor of caspase-8 to block caspase-8 activity [34]. Our findings showed that MMPP suppressed phosphorylation of IKKβ and IκBα, nuclear translocation of p50 and p65 and induced apoptotic cell death via regulating DR/caspase signaling. These data indicated that MMPP can inhibit colon cancer cell growth through induction of DR5 and DR6 via inhibition of IKKβ/NF-κB signaling *in vivo* and *in vitro*.

In addition, BHPB showed plausible in mutagenicity *in vivo* and chromosome damage *in vitro* while MMPP showed negative on predicted toxicities, and MMPP showed better aqueous solubility, human intestinal absorption and skin permeability on predicted ADME [35]. Therefore, MMPP exhibits potentiality of clinical trial for colorectal cancer treatment.

## MATERIALS AND METHODS

### Chemicals

MMPP was synthesized and identified as previously described [35]. MMPP is dissolved in DMSO in the present study.

### Materials

Caspase-3 and caspase-8 antibodies were purchased from Cell Signaling Technology Inc. (Beverly, MA, USA). Fas, DR3, DR4, DR5, DR6, IKKβ, p-IKKβ, IκBα, p-IκBα, p50, p65, Bcl-2, Bax, Histone-H1 and β-actin antibodies were purchased from Santa Cruz Biotechnology, Inc. (Santa Cruz, CA, USA). The cell culture materials were obtained from GIBGO^®^ of Introgen™ (Seoul, Korea).

### Cell culture

HCT116 and SW480 colon cancer or CCD-18Co colon epithelial cells were obtained from American Type Culture Collection (ATCC, Manassas, VA, USA). Both cells were from 3-passage stocks and were used before passage 30. The cells were tested to detect mycoplasma contamination using an e-MycoTM plus mycoplasma PCR detection kit (iNtRON Biotechnology, Seongnam, Gyeonggi, Korea). HCT116 and CCD-18Co cells were cultured in DMEM supplemented with 10% heat inactivated FBS, 100 units/mL penicillin and 100 μg/mL streptomycin. SW480 cells were cultured in RPMI supplemented with 10% heat inactivated FBS, 100 units/mL penicillin and 100 μg/mL streptomycin. Cells were maintained in an incubator within a humidified atmosphere of 5% CO_2_ at 37 °C.

### Cell viability assay

HCT116 and SW480 colon cancer or CCD-18Co colon epithelial cells were seeded in 96-well plates, next day were treated with MMPP (0-15 μg/mL) for 24 h. Cell viability assay was performed as previously described [35].

### Detection of Apoptosis

HCT116 and SW480 colon cancer cells were plated in 8-chamber slides, next day were treated with MMPP (0-15 μg/mL) for 24 h. TdT-mediated dUTP nick and labeling (TUNEL) assays were performed as previously described [35].

### Western blotting

HCT116 and SW480 colon cancer cells were treated with MMPP (0-15 μg/mL) for 24 h, then were homogenized with a protein extraction solution (PRO-PREP™, iNtRON Biotechnology, Seongnam, Gyeonggi, Korea), and lysed by 60 min incubation on ice. Western blotting was described elsewhere [36]. After a short washing in TBST, the membranes were immunoblotted with the following primary antibodies: caspase-3 and caspase-8 (1:1000 dilutions; Cell Signaling, Beverly, MA, USA) and Fas, DR3, DR4, DR5, DR6, IKKβ, p-IKKβ, IκBα, p-IκBα, p50, p65, Bcl-2, Bax, Histone-H1 and β-actin (1:1000 dilutions; Santa Cruz Biotechnology, Santa Cruz, CA, USA). The blots were performed using specific antibodies followed by second antibodies and visualization by chemiluminescence (ECL) detection system.

### Plasmid construction

C99S mutated IKKβ (C, a core amino acid of ATP binding site of IKKβ was replaced with an unrelated acid S) was generated by Cosmo Genetech (Seoul, Korea) and the mutants were checked by sequencing. HCT116 and SW480 colon cancer cells were plated in 6-well plates (2 x 10^5^ cells / well) and were transiently transfected with Mutated IKKβ (C99S), using the Lipofectamine 3000 reagent in OPTI-MEM for 48 h according to the manufacturer’s specification (Invitrogen, Carlsbad, CA, USA). The transfected cells were treated with MMPP (10 μg/mL) for 24 h and then were used for detecting cell viability and protein expression.

### Antitumor activity study in *in vivo* xenograft animal model

Seven-week-old male BALB/C nude mice were purchased from Orient-Bio (Seongnam, Gyunggi, Korea). The mice were maintained in accordance with the Korea Food and Drug Administration guidelines as well as the regulations for the care and use of laboratory animals of the animal ethics committee of Chungbuk National University (CBNU-278-11-01). HCT116 colon cancer cells were injected subcutaneous (1 x 10^7^ cells / 0.1 mL PBS/animals) with a 27-gauge needle into the right lower flanks in carrier mice. After 14 days, when the tumors had reached an average volume of 100-150 mm^3^, the tumor-bearing nude mice were intraperitoneally (i.p.) injected with MMPP (2.5 mg/kg and 5 mg/kg dissolved in 0.01% DMSO) twice per week for 3 weeks. The group treated with 0.01% DMSO was designated as the control. The weight and tumor volume of the animals were monitored twice per week. The tumor volumes were measured with vernier calipers and calculated by the following formula: (A x B^2^)/2, where A is the larger and B is the smaller of the two dimensions. At the end of the experiment, the animals were sacrificed. The tumors were separated from the surrounding muscles and dermis, excised and weighed.

### Immunohistochemistry

Animals were sacrificed by CO_2_, tumor tissues were collected. Immunohistochemistry assay was performed as described elsewhere [36]. Staining was carried out using antibodies against PCNA, p-IKKβ, active caspase-3, DR5 and DR6 (1:500, Abcam, Cambridge, UK).

### Pull-down assay

MMPP was conjugated with epoxy-activated Sepharose 6B. Pull-down assay was performed as described elsewhere [36]. MMPP (1 mg) was dissolved in 1mL of coupling buffer (0.1 M NaHCO_3,_ pH 13.0 containing 0.5 M NaCl). The proteins were resolved by SDS-PAGE followed by immunoblotting with antibodies against IKKβ (1:1000 dilutions, Santa Cruz Biotechnology, Santa Cruz, CA, USA).

### Docking experiment

Docking studies between IKKβ and MMPP was performed using Autodock VINA [37]. IKKβ crystal structure was used in the docking experiments and conditioned using AutodockTools by adding all polar hydrogen atoms. Three dimensional structures of IKKβ-DNA complexes were retrieved from the Protein Data Bank (PDB codes: 3BRT). Starting from the co-crystallized complexes, the IKKβ monomer chain (IKKβ from 3BRT), MMPP for docking were prepared using Maestro graphical interface. The grid box was centered on the IKKβ monomer and the size of the grid box was adjusted to include the whole monomer. Docking experiments were performed at various exhaustiveness values of the defaults: 16, 24, 32, 40 and 60. Molecular graphics for the best binding model was generated using Discovery Studio Visualizer 2.0.

### Statistical analysis

All statistical analysis was performed with GraphPad Prism 5 software (Version 5.0, GraphPad Software, Inc., San Diego, CA, USA). Group differences were analyzed using one-way ANOVA followed by Dunnett’s test or two-way analysis of variance followed by Tuckey’s test. All values are presented as mean ± S.D. Significance was set at *p* < 0.05 for all tests.

## Abbreviations

BHPB: (E)-2,4-bis(p-hydroxyphenyl)-2-butenal
CI: combination index
DR: death receptor
FADD: Fas-associated death domain
IKKβ: IkappaB kinase β
MMPP: (E)-2-methoxy-4-(3-(4-methoxyphenyl)prop-1-en-1-yl)phenol
NF-κB: nuclear factor kappa-light-chain-enhancer of activated B cells
